# Clinical efficacy of ultra-laser irradiation combined with gabapentin on elderly patients with cervical spondylotic radiculopathy

**DOI:** 10.3389/fneur.2025.1698860

**Published:** 2025-11-26

**Authors:** Zhou Yu, Haoliang Sun, Xin Liu

**Affiliations:** Department of Anesthesiology, The First People's Hospital of Fuyang, Hangzhou, Zhejiang, China

**Keywords:** elderly patients, cervical spondylotic radiculopathy, ultra-laser irradiation, gabapentin, pain relief

## Abstract

**Objective:**

Cervical spondylotic radiculopathy is the most prevalent form of cervical spondylosis. This study aimed to investigate the clinical efficacy of ultra-laser irradiation combined with gabapentin for treating elderly patients with cervical spondylotic radiculopathy, providing a basis for optimizing treatment strategies.

**Methods:**

In this single-blind randomized controlled trial, 160 elderly patients diagnosed at our hospital from March to June 2025 were assigned (1:1) to ultra-laser irradiation plus gabapentin or gabapentin alone (80 per group). Pain was assessed with the Numerical Rating Scale (NRS) and health status with the EQ-5D at baseline and after three courses of treatment (30 sessions in total). Adverse events were recorded.

**Results:**

Baseline NRS scores were similar between groups. After treatment, the between-group difference in NRS improvement was 1.90 points (95% CI 1.64–2.16; *p* < 0.001) favoring the combination. The between-group difference in EQ-5D change was 0.08 (95% CI 0.04–0.12; *p* < 0.001). Patients who experienced at least one adverse event accounted for 12.5% (10/80) in the combination group and 42.5% (34/80) in the gabapentin-only group (χ^2^ = 18.06, *p* < 0.001).

**Conclusion:**

Ultra-laser irradiation combined with gabapentin reduced pain, improved health-related quality of life, and was associated with fewer short-term adverse events compared with gabapentin alone. Confirmation with longer follow-up, sham-controlled designs, and cost-effectiveness evaluation is warranted.

## Introduction

Cervical spondylosis is a common and frequently occurring disease in clinical practice. In recent years, the incidence of cervical spondylosis has been rising, with cervical spondylotic radiculopathy accounting for 60% of all cases (1). According to relevant reports, the occurrence of cervical spondylotic radiculopathy is associated with the degeneration of cervical intervertebral disk and cervical spine joint. Such degeneration not only results in nerve root compression and stimulation but also induces symptoms related to neck, shoulders and upper limbs, including muscle weakness, pain and numbness. It’s reported that the theories related to the pathogenesis of cervical spondylotic radiculopathy include neuroinflammatory injury, autoimmune theory and mechanical compression, among which neuroinflammatory injury is the pathological basis and belongs to neuropathic pains (2–4).

At present, non-operative therapy, mainly including drugs, traction, physical therapy, nerve block, active prevention, is the major choice for treating cervical spondylotic radiculopathy. There are also a few cases choosing surgery. Unfortunately, surgical treatment is characterized by the demerits such as high recurrence rate, numerous complications, large trauma, destroyed stability of cervical vertebra structure, postoperative infection, the rejection, shedding and non-fusion of implants, and unstable curative effect (5). Similarly, western medicines, such as nonsteroidal anti-inflammatory drugs, antispasmodics and analgesics, have relatively large side effects. Overall, despite lots of therapeutic drugs and measures for cervical spondylotic radiculopathy, the curative effect is not satisfactory. Gabapentin, a derivative of γ-gamma-aminobutyric acid (GABA), is widely used in the treatment of neuropathic pain serving as a novel antiepileptic drug (6).

Ultra-laser pain therapy, also referred to as super-laser or high-intensity laser therapy in previous studies, is an emerging modality that has not yet been incorporated into international clinical guidelines or Cochrane systematic reviews. The analgesic and anti-inflammatory effects of high-intensity laser therapy are thought to be mainly mediated by photobiomodulation. Light energy in the 600–1,000 nm range is absorbed by mitochondrial cytochrome c oxidase (CCO), leading to increased ATP production, improved microcirculation, and decreased release of pro-inflammatory cytokines such as TNF-α and IL-6 (7–9). Nevertheless, it offers distinct advantages in pain management. The light of the ultra-laser pain therapeutic apparatus screened out by an optical filter has a wavelength of 600–1,000 nm and can penetrate 5 cm deep into tissues (10). Due to the advantages of no trauma, no pain and no collateral damage, the ultra-laser pain therapeutic apparatus has been widely applied in the treatment of pain and obtained an increasing attention (11). Ultra-laser can improve the permeability of blood vessel wall, reduce inflammatory exudation, and relieve congestion and edema by virtue of its photoelectron, electromagnetic wave and photochemical actions (9, 12). Nevertheless, there are few reports on the ultra-laser irradiation combined with gabapentin for treating cervical spondylotic radiculopathy. In this study, we investigated clinical effect of ultra-laser irradiation combined with gabapentin on elderly patients with cervical spondylotic radiculopathy, thereby providing a scientific basis for clinical practice.

## Methods

### General information

A total of 160 elderly patients with cervical spondylotic radiculopathy admitted to our hospital from March 2025 to June 2025 were randomly assigned to the observation or control group using a computer-generated random number table prepared by an independent statistician. All randomized participants completed the full treatment course and follow-up, and no dropouts or losses to follow-up occurred. This single-blind randomized controlled trial ensured allocation concealment through sealed, opaque, and sequentially numbered envelopes. Each envelope contained the group assignment card based on the pre-generated random sequence and was kept by an independent coordinator, who opened it only after patient enrollment. Investigators responsible for recruitment and treatment were blinded to the randomization list to maintain allocation integrity. The observation group received ultra-laser irradiation combined with gabapentin, while the control group received gabapentin alone. This study was approved by the Ethics Committee of our hospital and was registered with the ISRCTN registry (ISRCTN57406480) on March 13, 2025.

### Inclusion criteria

The included patients satisfied diagnostic criteria of cervical spondylotic radiculopathy in *Rehabilitation Guidelines for Diagnosis and Treatment of Cervical Spondylosis (2010)* published by Chinese Association of Rehabilitation Medicine; aged ≥ 60 years; had no other history of peripheral nerve disease; did not suffer from systemic diseases such as cardiovascular disease, respiratory disease, digestive disorder, and severe liver and kidney dysfunction; did not take opioids or non-steroidal analgesics, antidepressants and other antiepileptic drugs within 1 month (13, 14); understood and agreed to this study and signed the informed consent.

### Exclusion criteria

The excluded patients were combined with bone tuberculosis or severe osteoporosis; suffered from numbness, pain and fatigue of upper limbs caused by other lesions such as thoracic outlet syndrome, tennis elbow, carpal tunnel syndrome, cubital tunnel syndrome, scapulohumeral periarthritis, and tenosynovitis of biceps brachii; suffered from severe primary diseases such as cardiovascular diseases, cerebrovascular diseases, liver, kidney and hematopoietic system diseases; had poor adherence to treatment and incomplete clinical data, or underwent spinal fusion; had congenital musculoskeletal diseases, such as congenital torticollis and congenital myopathy (13, 15).

### Therapeutic methods

The ultra-laser irradiation was performed based on an ultra-laser pain therapeutic apparatus (also known as K2 infrared polarized light therapy apparatus, manufactured by Zhuhai Hema medical instrument co., LTD.) and a SG probe. Specifically, the patient was placed a supine position, with a low pillow under his shoulder to fully expose his neck. From 2.5 cm above the sternoclavicular joint, 1.5 cm outside the midline, the sternocleidomastoid muscle was pushed outward with the index finger, which could reach the C6 vertebra (located at the junction of the horizontal line of cricoid cartilage and the posterior edge of the ipsilateral muscle). Then, a SG lens was placed, with the maximum power output of 1,500 mW, focal diameter of 7 mm. Next, intermittent irradiation was performed 3 s, followed by 2 s of halt. The treatment was carried out for 10 min each time, once a day, for a total of 10 days (16). The ultra-laser device emits near-infrared light with a wavelength range of 600–1,000 nm. One course of treatment consisted of 10 consecutive daily sessions, and all outcomes were evaluated after three courses (30 sessions in total).

As for treatment with oral gabapentin capsules (Jiangsu Hengrui Pharmaceuticals Co., Ltd.; H20030662), the oral dose on the first day was 300 mg before bedtime; on the second day, 300 mg in the morning and evening; and on the third day, 300 mg in the morning, noon, and evening. After 3 days of treatment, the dose was gradually adjusted according to efficacy and tolerability, up to a maximum of 2,400 mg per day. The dose that effectively relieved pain was maintained. This titration schedule was based on previous randomized controlled trials in neuropathic pain. The patients in the two groups were treated once a day, with 10 times as a course of treatment (17, 18).

### Outcome measures

Numerical (pain) rating scale (NRS/NPRS) was employed for testing the severity of pain of the two group patients before treatment and after 3 courses of treatment and EuroQol-5 dimensions (EQ-5D) questionnaire for comparing their health status. After that, adverse reactions (gastrointestinal discomfort, dizziness, lethargy, edema, rash, ataxia, and fatigue) of patients occurring in the process of medication were recorded. Adverse events were summarized as the number of patients (n, %) experiencing each event. Patients could appear in multiple AE categories if they experienced more than one AE. The total AE rate represents the proportion of patients with at least one AE.

### Statistical methods

Statistical software SPSS20.0 was used for data processing. Continuous variables were expressed as mean ± standard deviation (SD), and the independent samples *t*-test was applied to compare these data between groups. Categorical variables were presented as frequencies and percentages, and comparisons were made using the chi-square test. The changes in NRS/NPRS and EQ-5D scores before and after treatment were analyzed with paired *t*-tests. Differences in post-treatment outcomes between the observation and control groups were assessed using analysis of covariance (ANCOVA), adjusting for baseline scores. The assumptions for ANCOVA (normality and homogeneity of variance) were checked and met. No missing data occurred, as all participants completed the study. For the two primary outcomes (NRS and EQ-5D), the Holm–Bonferroni correction was applied to control the overall significance level. *p* < 0.05 was considered statistically significant. For safety analysis, categorical comparisons were performed using the chi-square test based on the proportion of patients who experienced at least one adverse event in each group.

## Results

### Characteristics of the two group patients before treatment

**Figure 1:**
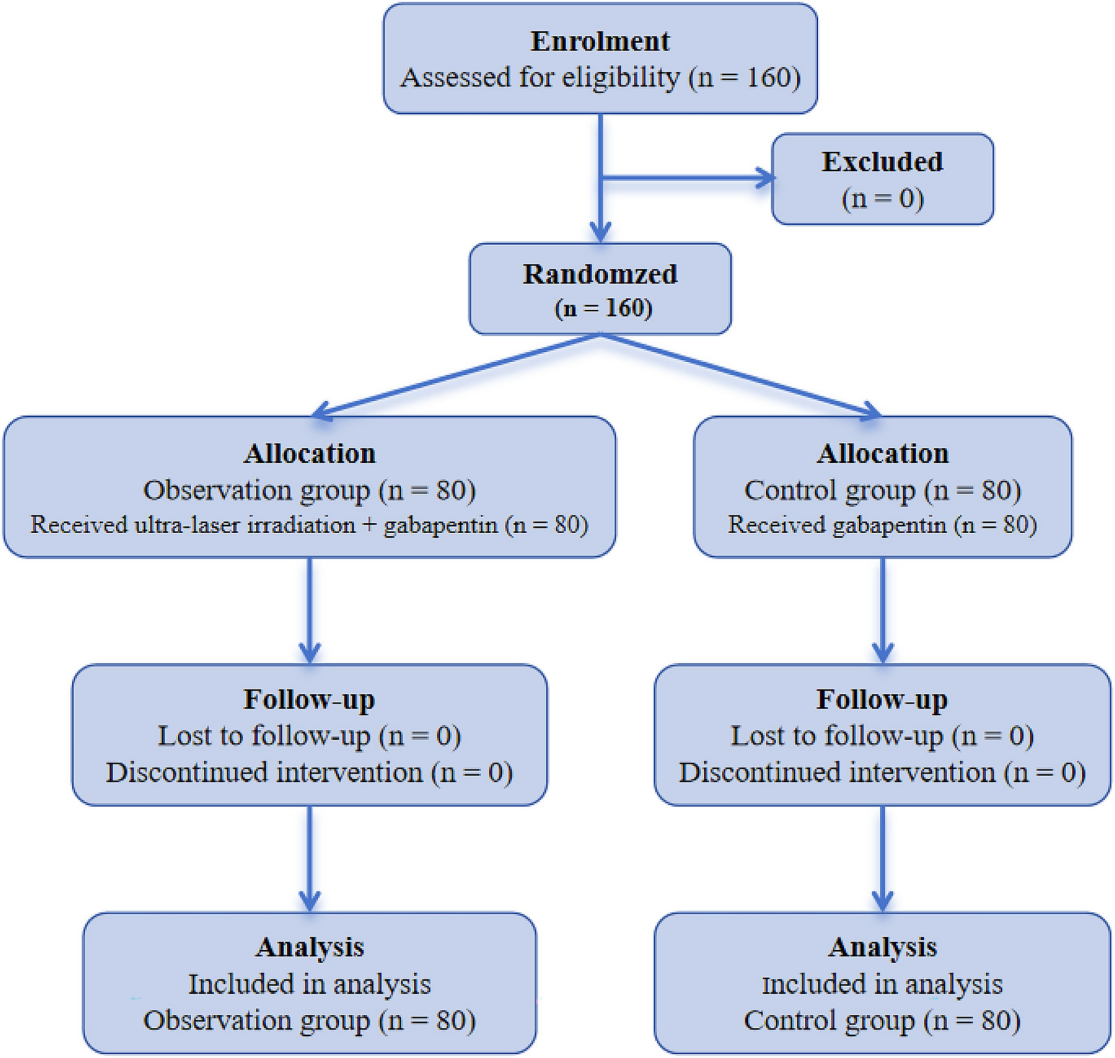
CONSORT flow diagram of patient enrollment and randomization in the trial of ultra-laser irradiation combined with gabapentin for cervical spondylotic radiculopathy.

The enrolment, allocation, follow-up, and analysis of patients are presented in Figure 1. A total of 160 patients were included in this study. Briefly, the observation group (*n* = 80) contained 32 males and 48 females, with the age ranging from 60 to 87 years (mean age: 66.39 ± 4.93 years); the control group (*n* = 80) consisted of 35 males and 45 females, aging from 60 to 79 years (mean age: 66.45 ± 4.39 years). There was no statistically significant difference in general information between the two groups (*p* > 0.05), indicating the comparability of general information for the two group patients (Table 1).

**Table 1:** Comparison of the general data between the two group patients.

	Observation group (*n* = 80)	Control group (*n* = 80)	χ^2^/*t*	*P*
Gender (%)			0.231	0.631
Male	32 (40.00)	35 (43.75)		
Female	48 (60.00)	45 (56.25)		
Age (year)	66.39 ± 4.93	66.45 ± 4.39	−0.085	0.933
Duration of disease (year)	7.70 ± 2.54	7.84 ± 2.87	−0.321	0.748
History of smoking (%)			1.823	0.177
No	58 (72.50)	50 (62.50)		
Yes	22 (27.50)	30 (37.50)		
Occupation (%)			0.245	0.620
Unemployed	53 (66.25)	50 (62.50)		
Retired	27 (33.75)	30 (37.50)		
History of previous medication (%)			0.249	0.618
No	54 (67.50)	51 (63.75)		
Yes	26 (32.50)	29 (36.25)		

### Comparison of pain outcomes between the two groups

As shown in Table 2, the difference was not significant in NRS/NPRS scores between the observation group (5.38 ± 0.54) and control group (5.36 ± 0.64) before treatment (*p* > 0.05). After treatment, the NRS/NPRS score in the observation group was (2.26 ± 0.59) and that in the control group was (4.15 ± 0.58). Compared with that before treatment, the NRS/NPRS change was (3.11 ± 0.80) in the observation group and (1.21 ± 0.84) in the control group [mean difference after adjustment was 1.90 (95% confidence interval [CI] = 1.64–2.16); *p* < 0.001]. The NRS/NPRS score of the two groups after treatment was significantly different from that before treatment (*p* < 0.05). Besides, the observation group presented much lower NRS/NPRS score than the control group after treatment, with a significant difference (*p* < 0.05).

**Table 2:** Changes of NRS/NPRS score in two groups before and after treatment.

	Observation group (*n* = 80)		Control group (*n* = 80)	
	Before treatment	After 3 courses of treatment	Before treatment	After 3 courses of treatment
NRS/NPRS				
Mean ± standard deviation	5.38 ± 0.54	2.26 ± 0.59^*#^	5.36 ± 0.64	4.15 ± 0.58^#^
NRS/NPRS changes				
Variation value	–	3.11 ± 0.80^*^	–	1.21 ± 0.84
Mean difference (95% CI)	–	1.90 (1.64–2.16)	–	–
*p* value		<0.001		

^*^P < 0.05 vs. control group; ^#^*P* < 0.05 vs. before treatment; NRS/NPRS, numerical (pain) rating scale; CI, confidence interval.

### Health status of the two group patients

As shown in Table 3, the EQ-5D scores of the two groups improved both before treatment and after three courses of treatment. In comparison with the control group, the improvement of EQ-5D score in the observation group after 3 courses of treatment was more significant [mean difference (95%CI) = 0.08 (0.04–0.12), *p* < 0.001].

**Table 3:** Changes of EQ-5D scores before and after treatment in two groups.

	Observation group (*n* = 80)		Control group (*n* = 80)	
	Before treatment	After 3 courses of treatment	Before treatment	After 3 courses of treatment
EQ-5D score				
Mean ± standard deviation	0.602 ± 0.136	0.763 ± 0.120^*#^	0.595 ± 0.114	0.676 ± 0.110^#^
EQ-5D changes				
Variation value	–	0.161 ± 0.138^*^	–	0.081 ± 0.108
Mean difference (95% CI)	–	0.08 (0.04–0.12)	–	–
*P* value		<0.001		

^*^P < 0.05 vs. control group; ^#^*P* < 0.05 vs. before treatment; EQ-5D, EuroQol-five dimensions; CI, confidence interval.

Effect size and power for the two primary outcomes are summarized in Supplementary Table S1, which demonstrates that the treatment effects are both statistically significant and clinically meaningful.

### Incidence of adverse events in the two groups

Adverse reactions of the two group patients after treatment were shown as follows. In the observation group, the maximum oral dose of gabapentin was not more than 2,400 mg/day, and there were only 7 cases of fatigue (8.8%) and 4 cases of nausea (5.0%). In the control group, the maximum oral dose of gabapentin did not exceed 2,400 mg/day, and there were 10 cases of dizziness (12.5%), 8 cases of lethargy (10.0%), 12 cases of ataxia (15.0%), 12 cases of fatigue (15.0%), and 7 cases of nausea (8.8%). As for other patients, they did not occur obvious adverse events. The difference in the number of adverse reactions after treatment was statistically significant between the two groups (χ^2^ = 18.056, *p* < 0.001) (Table 4).

**Table 4:** Comparison of adverse event rates between the two groups.

Grouping	*n*	Dizziness	Lethargy	Fatigue	Nausea	Ataxia	Patients with ≥1 adverse event, *n* (%)	χ^2^	*P*
Observation group	80	0 (0)	0 (0)	7 (8.8)	4 (5.0)	0 (0)	10 (12.5)	18.056	<0.001
Control group	80	10 (12.5)	8 (10.0)	12 (15.0)	7 (8.8)	12 (15.0)	34 (42.5)		

## Discussion

This randomized controlled trial demonstrated that ultra-laser irradiation combined with gabapentin significantly reduced pain intensity and improved health-related quality of life compared with gabapentin alone in elderly patients with cervical spondylotic radiculopathy (CSR). The improvements in NRS (1.9 points) and EQ-5D (0.08) both exceeded the commonly accepted minimal clinically important difference thresholds, suggesting that the observed effects were clinically meaningful.

The superior efficacy of the combination therapy may be explained by complementary mechanisms. High-intensity near-infrared laser therapy exerts photobiomodulation effects through mitochondrial cytochrome c oxidase activation, leading to enhanced ATP production, improved microcirculation, and suppression of pro-inflammatory mediators such as TNF-α and IL-6 (19). These cellular effects can reduce neuroinflammatory sensitization and restore local metabolic balance, thereby alleviating neuropathic pain. Meanwhile, gabapentin modulates the α₂*δ* subunit of voltage-gated calcium channels, decreasing excitatory neurotransmitter release and neuronal hyperexcitability. The concurrent modulation of both peripheral and central pain pathways likely contributes to the synergistic analgesic effect observed.

Our findings are consistent with previous studies showing that photobiomodulation combined with pharmacologic therapy enhances pain relief compared with either modality alone. Abdel-Wahhab KG et al. demonstrated that low-level laser therapy combined with gabapentin produced superior analgesia in diabetic neuropathy models (20), while Farazi N et al. reported enhanced anti-inflammatory and neuromodulatory benefits with multimodal phototherapy approaches (21). Together, these results support the rationale for integrating ultra-laser irradiation with gabapentin in neuropathic pain management. In addition to improved efficacy, the combination therapy showed a lower incidence of adverse events. The reduced rate of dizziness, lethargy, and ataxia may reflect lower effective doses of gabapentin achieved through adjunctive laser therapy. Given its noninvasive nature, absence of systemic toxicity, and favorable tolerability, ultra-laser irradiation represents a clinically acceptable complementary intervention for elderly patients with CSR.

There are several limitations to this study. Blinding was not feasible because the ultra-laser emits visible light and produces mild warmth, which could allow participants to identify the intervention and may lead to potential expectation bias despite assessor blinding. In addition, the present study evaluated only short-term outcomes and did not include a sham-laser control group, which may limit interpretation of the treatment-specific effects. The sample was from a single center, and cost-effectiveness was not assessed. Future multicenter randomized trials with longer follow-up and economic evaluation are warranted to further validate and extend these findings.

## Conclusion

To sum up, the combination of ultra-laser irradiation and gabapentin therapy effectively reduces clinical symptoms and alleviates disease severity in elderly patients with cervical spondylotic radiculopathy. Furthermore, such a combination therapy is highly effective, provides significant pain relief, and has tolerable adverse effects, making it highly valuable for clinical application and worthy of further investigation.

## Data Availability

The original contributions presented in the study are included in the article/supplementary material, further inquiries can be directed to the corresponding author.
