# Supplementary material for: Clinical efficacy of ultra-laser irradiation combined with gabapentin on elderly patients with cervical spondylotic radiculopathy
Source: Front Neurol. 2025 Nov 26;16:1698860. doi: 10.3389/fneur.2025.1698860 (PMC12710229; doi:10.3389/fneur.2025.1698860)
Supplement: Supplementary file 1 [file Table_1.docx]

Supplementary Table S1. Post-hoc effect size and power analysis for the primary outcomes

| Outcome | ΔNRS | ΔEQ-5D |
| --- | --- | --- |
| Observation group (Mean ± SD) | −3.11 ± 0.80 | 0.161 ± 0.138 |
| Control group (Mean ± SD) | −1.21 ± 0.84 | 0.081 ± 0.108 |
| Mean difference (95% CI) | −1.90 (−2.16 to −1.65) | 0.08 (0.04 to 0.12) |
| Cohen’s d | 2.33 | 0.65 |
| Power | 1.000 | 0.982 |

Note: NRS, numerical rating scale; EQ-5D, EuroQol-five dimensions; CI, confidence interval; MCID, minimal clinically important difference; Cohen’s d, standardized effect size.
